# Relapse following FLT3 inhibitor cessation in *FLT3*-ITD-positive AML: lessons from two clinical cases

**DOI:** 10.1007/s00277-026-06776-w

**Published:** 2026-02-04

**Authors:** Julia-Annabell Georgi, Christoph Röllig, Johannes Schetelig, Christian Thiede, Sascha Brückmann, Martin Bornhäuser, Jan Moritz Middeke

**Affiliations:** 1 Medical Department 1, Faculty of Medicine and University Hospital Carl Gustav Carus, University of Technics Dresden, Dresden, Germany; 2 DKMS Clinical Trials Unit, Dresden, Germany; 3 AgenDix GmbH, Dresden, Germany; 4 Institute of Pathology, Faculty of Medicine and University Hospital Carl Gustav Carus, University of Technics Dresden, Dresden, Germany; 5 National Center for Tumor Diseases (NCT), Dresden, Germany

**Keywords:** *FLT3*-ITD AML, *FLT3* inhibitor, Post-transplant maintenance, Gilteritinib, Relapse, Measurable residual disease (MRD)

## Abstract

The clinical success of *FLT3* inhibitors has led to their steadily increasing use in the treatment of acute myeloid leukemia (AML), both in the relapsed/refractory setting and as post-transplant maintenance. Despite their expanding application, there is currently no guidance on the optimal duration of therapy or the feasibility of discontinuation. In the maintenance context, current practice is largely based on trial protocols with predefined treatment periods, yet relapses after cessation have been documented. Similarly, in the relapsed/refractory setting, the management of long-term responders to *FLT3*-directed monotherapy lacks evidence-based guidance.

We report two cases of *FLT3*-ITD AML patients with relapse after discontinuation of prolonged *FLT3* inhibitor therapy, despite sustained remission prior to withdrawal. As such scenarios remain insufficiently characterized in the literature, these case vignettes are presented to highlight the unresolved challenge of defining the appropriate duration of *FLT3* inhibitor therapy and to underscore the need for systematic evaluation to establish evidence-based strategies for safe discontinuation or extended administration.

## Introduction

The introduction of *FLT3* inhibitors has reshaped the therapeutic landscape of acute myeloid leukemia (AML), especially in patients harboring *FLT3*-internal tandem duplication (*FLT3*-ITD) mutations [1]. *FLT3*-ITD AML, typically associated with high relapse rates and poor prognosis, is now showing improved outcomes with *FLT3* inhibition, including deep and durable remissions in some cases. Even in the relapsed or refractory setting, *FLT3* inhibitor monotherapy can reinduce remission [2, 3]. These improvements raise new questions regarding *FLT3*-ITD AML management, including the challenge of defining the appropriate duration of *FLT3* inhibitor therapy for long-term disease control. This issue holds significant relevance for clinical practice, as *FLT3* inhibitors are used across multiple settings, including both relapse treatment and maintenance therapy in post-transplant and non-transplant contexts.

Gilteritinib is the only *FLT3* inhibitor approved for monotherapy in relapsed/refractory (r/r) *FLT3*-mutated AML and is routinely continued until progression or as bridge to transplant [2]. However, in patients achieving long-term remissions without subsequent allogeneic hematopoietic cell transplantation (alloHCT) [4], the optimal duration of therapy remains undefined, and it is unclear whether treatment can be safely discontinued at any point.

In the post-transplant setting, *FLT3* inhibitors are widely used as maintenance therapy. In the absence of formal guidelines, current practice is based on clinical trial protocols employing a two-year maintenance course [5, 6]. Relapse following the discontinuation of therapy at these fixed time points has been reported [6], raising questions whether this duration is sufficient for all patients or whether some may benefit from prolonged maintenance.

We highlight the clinical challenge of defining the optimal duration of *FLT3* inhibition by presenting two *FLT3*-ITD AML patients with relapse occurring after treatment discontinuation despite prior prolonged remissions. Written informed consent for publication of anonymized data was obtained in accordance with the Declaration of Helsinki.

## Case vignettes

### Case 1

A 72-year-old male was diagnosed in October 2019 with AML harboring *FLT3*-ITD (allelic ratio 0.89), *FLT3*-TKD and an *NPM1* mutation with a normal karyotype, corresponding to ELN 2017 intermediate risk [7]. At diagnosis, peripheral blood showed 96% blasts, marked hyperleukocytosis, anemia, thrombocytopenia and elevated LDH. Aside from atrial fibrillation, no significant comorbidities were present.

The patient received one induction cycle with daunorubicin, cytarabine and midostaurin, achieving complete remission with incomplete hematologic recovery. In line with institutional standards at the time, he proceeded to alloHCT (9/10 HLA-mismatched unrelated donor) in December 2019. Pre-transplant evaluation demonstrated persistent measurable residual disease (MRD), with an *NPM1/ABL1* level [8] of 0.05%. Conditioning was performed with sequential reduced-intensity conditioning regimen (FLAMSA-RIC); graft-versus-host disease (GvHD) prophylaxis included cyclosporine A, mycophenolate mofetil, and anti-thymocyte globulin.

Early post-transplant follow-up showed progressive disease, evidenced by mixed donor chimerism and rising NPM1 MRD, prompting early cessation of immunosuppression. In March 2020, bone marrow (BM) examination confirmed hematologic relapse. Molecular analysis identified re-emergence of *FLT3*-ITD and TKD mutations. Salvage therapy with gilteritinib (120 mg/day) was initiated, leading to rapid clearance of peripheral blasts.

This response allowed for a second alloHCT from a haploidentical related donor in May 2020. Pre-transplant MRD assessment after one cycle of gilteritinib demonstrated an *NPM1* transcript level of 77%. Conditioning included intensified RIC with fludarabine/8Gy TBI; GvHD prophylaxis consisted of post-transplant cyclophosphamide, mycophenolate mofetil, and tacrolimus. By July 2020, molecular remission (*NPM1/ABL1* < 0.001%) was documented in the BM.

Gilteritinib maintenance (120 mg/day) was initiated around day + 40 post-transplant. Due to infections and declining renal function, the dose was intermittently reduced to 80 mg/day. NPM1 MRD remained undetectable throughout monitoring. In August 2022, after two years of maintenance therapy and in the absence of detectable MRD, gilteritinib was discontinued in accordance with the protocol used in the MORPHO trial [6].

In November 2022, MRD monitoring showed rising *NPM1/ABL1* (217%), indicating imminent hematologic relapse. In addition, molecular testing confirmed reappearance of both *FLT3*-ITD (VAF 2%) and *FLT3*-TKD (VAF 2.4%) mutations. Gilteritinib was reinitiated at 120 mg/day and was again well tolerated. In September 2023, due to persistent MRD positivity and in the absence of GvHD, one donor lymphocyte infusion (DLI) was administered (1 × 10⁶ CD3⁺ cells/kg). Ongoing therapy has been associated with a continuous decline in NPM1 MRD burden. By April 2025, the *NPM1* transcript level had declined to 0.001%, indicating deep molecular response. Gilteritinib remains well tolerated at full dose to date.

**Fig. 1:**
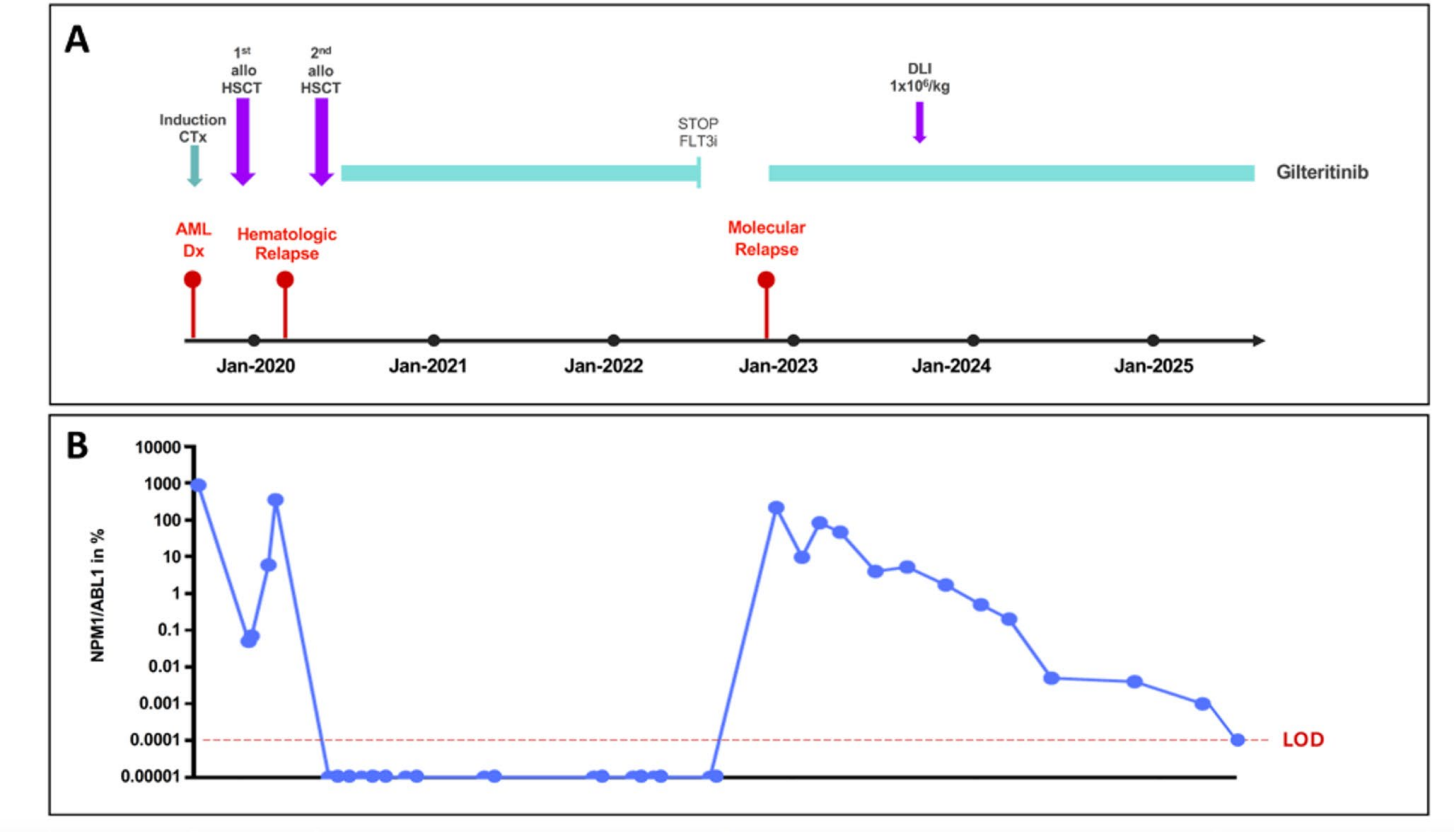
**Clinical course and molecular monitoring in patient 1.** Clinical timeline (A) and molecular surveillance based on NPM1/ABL1 transcript levels (B). *Abbreviations:* alloHCT, allogeneic hematopoietic cell transplantation; CTx, chemotherapy; DLI, donor lymphocyte infusion; Dx, diagnosis; FLT3i, *FLT3* inhibitor; LOD, level of detection

A schematic overview of the patient’s clinical course and molecular surveillance is provided in Fig. 1.

### Case 2

A 53-year-old female was diagnosed with AML in March 2017. BM examination showed normal karyotype and a high allelic ratio *FLT3*-ITD mutation (0.94), categorizing the case as adverse risk according to ELN 2017 [7]. No significant comorbidities were present.

Induction with daunorubicin/cytarabine failed to induce complete remission (CR). Salvage therapy was initiated within the MIRROS trial using idarubicin and cytarabine ± idasanutlin, an MDM2 antagonist [9]. CR was documented on BM by day 15.

In May 2017, she underwent alloHCT from a 10/10 HLA-matched unrelated donor with myeloablative conditioning (busulfan/cyclophosphamide). GvHD prophylaxis included cyclosporine A and methotrexate. She showed prompt engraftment, full donor chimerism and no significant acute GvHD. Post-transplant, declining donor chimerism was observed, prompting early immunosuppression taper. However, in October 2017, the patient experienced hematologic relapse with 70% BM blasts and reappearance of the *FLT3*-ITD mutation. She was enrolled in the ADMIRAL trial [2] and received gilteritinib at 120 mg/day, resulting in rapid hematologic response and complete donor chimerism. Gilteritinib was continued as monotherapy. During follow-up, she developed moderate chronic GvHD affecting the oral mucosa, skin, and lungs.

Following sustained remission for over five years, with ongoing complete donor chimerism and active chronic GvHD, gilteritinib was discontinued in October 2023 after comprehensive clinical and molecular assessment.

In early 2025, the patient developed back pain accompanied by progressive gait disturbance. Spinal MRI revealed an intraspinal mass at L5/S1 with epidural compression. PET imaging demonstrated mild hypermetabolic activity; histopathology of the biopsied lesion confirmed infiltration by an undifferentiated hematologic neoplasm. Definitive classification was achieved through molecular analysis, which detected the original *FLT3*-ITD at a variant allele frequency (VAF) of 47%, consistent with late extramedullary AML relapse.

At the same time, BM biopsy continued to show hematologic CR and complete donor chimerism. However, amplicon-based ultradeep sequencing detected the known *FLT3*-ITD [10] at a low VAF of 0.067%, suggesting early molecular recurrence or persistence.

Due to extramedullary relapse, gilteritinib was reinitiated in April 2025 at 120 mg/day resulting in rapid symptom resolution and reduction of the lesion on imaging. Consolidative radiotherapy of the lumbosacral spine was initiated with a total dose of 36 Gy. Gilteritinib remains well tolerated and is being continued.

**Fig. 2:**
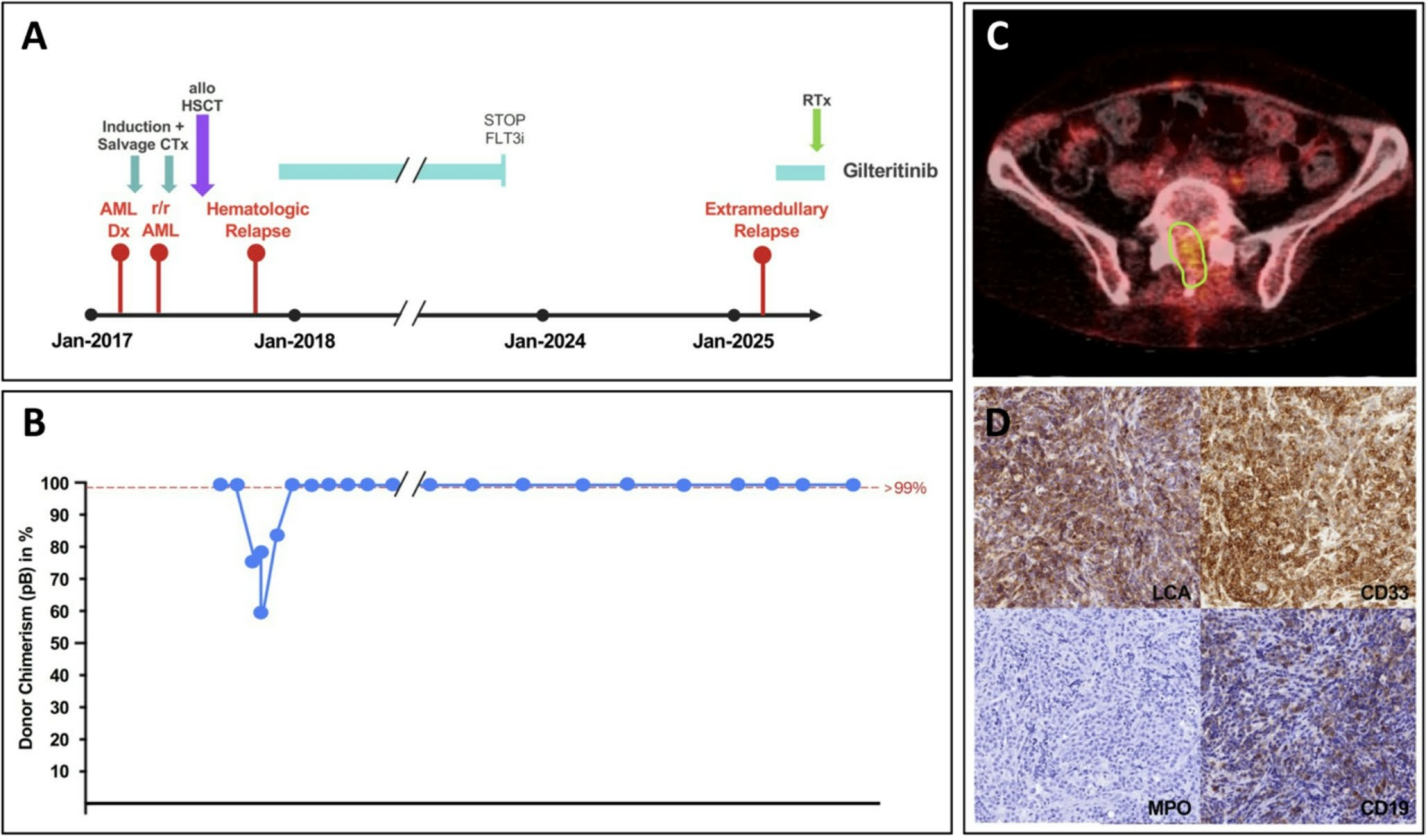
**Clinical course, donor chimerism monitoring, and diagnostics of extramedullary relapse in patient 2.** Clinical timeline (**A**), donor chimerism monitoring (**B**), and diagnostic work-up of extramedullary relapse, including PET imaging (**C**) and histopathological analysis of the intraspinal lesion (**D**) characterized by expression of CD45 (LCA), CD33, and CD19, with absent expression of MPO. *Abbreviations*: alloHCT, allogeneic hematopoietic cell transplantation; CTx, chemotherapy; DLI, donor lymphocyte infusion; Dx, diagnosis; FLT3i, *FLT3* inhibitor; LCA, leucocyte common antigen; LOD, level of detection; MPO, myeloperoxidase; pB, peripheral blood; PET, positron emission tomography; r/r, relapsed/refractory; RTx, radiotherapy

Figure 2 provides a schematic overview of the patient’s clinical course and chimerism monitoring, as well as imaging and histopathological work-up of the extramedullary relapse.

## Discussion

In the absence of guidance on the appropriate duration of application or feasibility of discontinuing *FLT3* inhibitor therapy, current treatment decisions are largely based on individual clinical judgment and institutional practice. This uncertainty poses a relevant clinical challenge: while prolonged or indefinite *FLT3* inhibitor therapy may represent overtreatment for some patients and cause substantial socioeconomic burden, discontinuation may expose others to the risk of relapse. As illustrated by the case vignettes, relapse following cessation of *FLT3* inhibitor therapy can occur even after prolonged remission. Similar observations have also been reported in clinical trials. For example, cases of relapse after cessation of *FLT3* inhibitor maintenance post-transplant have been observed in the MORPHO trial, raising concerns that a fixed two-year duration may be insufficient to achieve sustained disease control in some patients [6]. In the r/r setting, long-term follow-up from the ADMIRAL trial identified a subset of patients who remained in remission beyond two years on continued gilteritinib treatment [4]. However, data on long-term responders are limited, and neither structured discontinuation strategies nor relapse patterns after gilteritinib cessation have been systematically evaluated. Establishing a robust evidence base should be a primary objective, as it provides the foundation for individualized relapse risk assessment and guidance on *FLT3* inhibitor duration.

In the context of relapse prediction and individualized therapeutic decisions, measurable residual disease (MRD) may serve as a promising tool. While *FLT3*-ITD based MRD assessment would offer the highest specificity, current methodologies for detecting *FLT3*-ITD with sufficient sensitivity and reliability remain technically challenging and lack standardization. The MORPHO trial attempted risk stratification based on pre-transplant MRD assessment using NGS for *FLT3*-ITD detection [6], yet relapse occurred in both MRD positive and negative patients, suggesting limited predictive value [2]. Longitudinal post-transplant MRD monitoring may add important value by enabling dynamic risk assessment and guiding timely therapeutic interventions. In clinical practice, the implementation of such highly sensitive molecular assays remains technically challenging and heterogeneous across institutions. Consequently, results obtained with different MRD assessment methods should be interpreted with due consideration when informing therapeutic decisions. Additionally, biological factors such as clonal evolution, including *FLT3-*ITD negative relapse, further complicate the interpretation of MRD results [11]. Importantly, MRD negativity - even when assessed with highly sensitive markers, as illustrated in the first case - does not necessarily indicate complete disease eradication. Despite these limitations, longitudinal MRD monitoring remains clinically valuable, as it enables early relapse detection and timely treatment reinitiation.

The second case highlights additional limitations of BM-centered follow-up. Sanctuary sites, characterized by microenvironmental protection and limited *FLT3* inhibitor penetration, may enable clonal escape despite sustained remission within the BM [12, 13]. Extramedullary relapse, reported in up to 15% of *FLT3*-ITD AML posttransplant [14, 15], thus remains a relevant concern even with targeted therapies.

Biologically, both cases support the understanding that *FLT3* inhibitors, while effective at suppressing proliferative leukemic cells, are less capable of eliminating the quiescent leukemic stem cell reservoir that can drive eventual relapse [16, 17]. Emerging data suggest that *FLT3* mutations are not necessarily present in the earliest leukemic stem cells but may arise in more differentiated subclones during disease evolution [18]. This may indicate that clonal architecture - specifically whether *FLT3*-ITD is part of the founding clone - could influence relapse risk under *FLT3*-directed therapy.

In the post-transplant setting, the potential impact of immunologic factors such as the graft-versus-leukemia (GvL) effect and the presence or absence of GvHD must also be considered, as they influence individual relapse risk and may modify both the necessity and benefit of continued *FLT3* inhibitor therapy. Preclinical data indicate that *FLT3* inhibitors enhance GvL effects by preserving antileukemic effector pathways and CD8⁺ T cell function [19, 20], supporting the rationale for prolonged administration.

Beyond biological considerations, practical clinical factors also play a role. For patients ineligible for further salvage therapy, prolonged *FLT3* inhibitor treatment may represent a reasonable option. The tolerability profile of second-generation *FLT3* inhibitors, such as gilteritinib [21], supports their feasibility for long-term use. However, an approach based on indefinite therapy is unlikely to be broadly feasible due to financial, logistical, and psychosocial constraints.

Given these complexities, the optimal duration of *FLT3* inhibitor therapy - both post-transplant and in the r/r setting - remains an open question. As it becomes increasingly evident that fixed treatment durations may not adequately account for the biological and clinical heterogeneity of *FLT3*-ITD AML, robust follow-up and real-world data are required to develop evidence-based strategies for safe discontinuation or extended therapy.

## Data Availability

No datasets were generated or analysed during the current study.

## References

[1] Daver N, Schlenk RF, Russell NH, Levis MJ (2019) Targeting FLT3 mutations in AML: review of current knowledge and evidence. Leukemia 33:299–312 30651634 10.1038/s41375-018-0357-9 PMC6365380

[2] Perl AE, Martinelli G, Cortes JE et al (2019) Gilteritinib or chemotherapy for relapsed or refractory FLT3-Mutated AML. N Engl J Med 381:1728–1740 31665578 10.1056/NEJMoa1902688

[3] Cortes JE, Khaled S, Martinelli G et al (2019) Quizartinib versus salvage chemotherapy in relapsed or refractory FLT3-ITD acute myeloid leukaemia (QuANTUM-R): a multicentre, randomised, controlled, open-label, phase 3 trial. Lancet Oncol 20:984–997 31175001 10.1016/S1470-2045(19)30150-0

[4] Perl AE, Larson RA, Podoltsev NA et al (2022) Follow-up of patients with R/R FLT3-mutation-positive AML treated with gilteritinib in the phase 3 ADMIRAL trial. Blood 139:3366–3375 35081255 10.1182/blood.2021011583 PMC9197557

[5] Burchert A, Bug G, Fritz LV et al (2020) Sorafenib maintenance after allogeneic hematopoietic stem cell transplantation for acute myeloid leukemia with FLT3-Internal tandem duplication mutation (SORMAIN). J Clin Oncol 38:2993–3002 32673171 10.1200/JCO.19.03345

[6] Levis MJ, Hamadani M, Logan B et al (2024) Gilteritinib as Post-Transplant maintenance for AML with internal tandem duplication mutation of FLT3. J Clin Oncol 42:1766–1775 38471061 10.1200/JCO.23.02474 PMC11095884

[7] Döhner H, Estey E, Grimwade D et al (2017) Diagnosis and management of AML in adults: 2017 ELN recommendations from an international expert panel. Blood 129:424–447 27895058 10.1182/blood-2016-08-733196 PMC5291965

[8] Gorello P, Cazzaniga G, Alberti F et al (2006) Quantitative assessment of minimal residual disease in acute myeloid leukemia carrying nucleophosmin (NPM1) gene mutations. Leukemia 20:1103–1108 16541144 10.1038/sj.leu.2404149

[9] Montesinos P, Beckermann BM, Catalani O et al (2020) MIRROS: a randomized, placebo-controlled, phase III trial of cytarabine ± idasanutlin in relapsed or refractory acute myeloid leukemia. Future Oncol 16:807–815 32167393 10.2217/fon-2020-0044

[10] Blätte TJ, Schmalbrock LK, Skambraks S et al (2019) GetITD for FLT3-ITD-based MRD monitoring in AML. Leukemia 33:2535–2539 31089248 10.1038/s41375-019-0483-z PMC8075860

[11] Schmalbrock LK, Dolnik A, Cocciardi S et al (2021) Clonal evolution of acute myeloid leukemia with FLT3-ITD mutation under treatment with midostaurin. Blood 137:3093–3104 33598693 10.1182/blood.2020007626 PMC8233666

[12] Seo S, Kami M, Honda H et al (2000) Extramedullary relapse in the so-called ‘sanctuary’ sites for chemotherapy after donor lymphocyte infusion. Bone Marrow Transpl 25:226–227 10.1038/sj.bmt.1702116 10673689

[13] Ambinder AJ, Levis M (2021) Potential targeting of FLT3 acute myeloid leukemia. Haematologica 106:671–681 32703795 10.3324/haematol.2019.240754 PMC7927884

[14] Jabbour E, Guastad Daver N, Short NJ et al (2017) Factors associated with risk of central nervous system relapse in patients with non-core binding factor acute myeloid leukemia. Am J Hematol 92:924–928 28556489 10.1002/ajh.24799 PMC5901967

[15] Kouidri K, Acker F, Toenges R et al (2024) Central nervous system relapse after allogeneic HCT in FLT3-mutated AML. Ann Hematol 103:5387–5393 39589497 10.1007/s00277-024-06106-y PMC11695448

[16] Parmar A, Marz S, Rushton S et al (2011) Stromal niche cells protect early leukemic FLT3-ITD + progenitor cells against first-generation FLT3 tyrosine kinase inhibitors. Cancer Res 71:4696–4706 21546568 10.1158/0008-5472.CAN-10-4136

[17] Chang YT, Hernandez D, Alonso S et al (2019) Role of CYP3A4 in bone marrow microenvironment-mediated protection of FLT3/ITD AML from tyrosine kinase inhibitors. Blood Adv 3:908–916 30898762 10.1182/bloodadvances.2018022921 PMC6436013

[18] Pollard JA, Alonzo TA, Gerbing RB et al (2006) FLT3 internal tandem duplication in CD34+/CD33- precursors predicts poor outcome in acute myeloid leukemia. Blood 108:2764–2769 16809615 10.1182/blood-2006-04-012260 PMC1895585

[19] Mathew NR, Baumgartner F, Braun L et al (2018) Sorafenib promotes graft-versus-leukemia activity in mice and humans through IL-15 production in FLT3-ITD-mutant leukemia cells. Nat Med 24:282–291 29431743 10.1038/nm.4484 PMC6029618

[20] Zhang Z, Hasegawa Y, Hashimoto D et al (2022) Gilteritinib enhances graft-versus-leukemia effects against FLT3-ITD mutant leukemia after allogeneic hematopoietic stem cell transplantation. Bone Marrow Transpl 57:775–780 10.1038/s41409-022-01619-4 35228711

[21] Perl AE, Altman JK, Cortes J et al (2017) Selective Inhibition of FLT3 by gilteritinib in relapsed or refractory acute myeloid leukaemia: a multicentre, first-in-human, open-label, phase 1–2 study. Lancet Oncol 18:1061–1075 28645776 10.1016/S1470-2045(17)30416-3 PMC5572576

